# Antimicrobial activity of spiculisporic acid isolated from endophytic fungus *Aspergillus cejpii* of *Hedera helix* against MRSA

**DOI:** 10.1007/s42770-023-01224-7

**Published:** 2024-01-17

**Authors:** Sarah Osama, Moshera El Sherei, Dalia A. Al-Mahdy, Mokhtar Bishr, Osama Salama, Marwa M. Raafat

**Affiliations:** 1 Pharmacognosy and Medicinal Plants Department, Faculty of Pharmacy, Future University in Egypt, Cairo, Egypt; 2 Department of Pharmacognosy, Faculty of Pharmacy, Cairo University, Cairo, Egypt; 3 Department of Pharmacognosy, Faculty of Pharmacy, Modern University for Technology and Information (MTI), Cairo, Egypt; 4 Arab Company for Pharmaceuticals and Medicinal Plants (Mepaco), Cairo, Egypt; 5 Microbiology and Immunology Department, Faculty of Pharmacy, Future University in Egypt, Cairo, 11835 Egypt

**Keywords:** *Staphylococcus aureus*, *Pseudomonas aeruginosa*, *Acinetobacter baumannii*, NMR, GC-MS, Bioguided fractionation, Antimicrobial susceptibility, Spiculosporic acid

## Abstract

The surge in multidrug-resistant pathogens worldwide has jeopardized the clinical efficiency of many current antibiotics. This problem steered many researchers in their quest to discover new effective antimicrobial agents from natural origins including plants or their residing endophytes. In this work, we aimed to identify the endophytic fungi derived from *Hedera helix* L. and investigate their potential antimicrobial activity. Bioguided fractionation approach was conducted to isolate the pure compounds from the most active fungal fraction. Out of a total of six different isolated endophytic fungal strains, only *Aspergillus cejpii* showed the highest activity against all tested microbial strains. The most active fraction was the dichloromethane/methanol fraction (DCM:MeOH), where it showed significant activity against *Escherichia coli*, *Pseudomonas aeruginosa*, *Staphylococcus aureus*, *Serratia marcescens*, *Acinetobacter baumannii*, *Salmonella typhi*, and three drug-resistant clinical isolate strains including Methicillin-resistant *Staphylococcus aureus* (MRSA, H1), *Pseudomonas aeruginosa* (PS 16), and *Acinetobacter baumannii* (ACT 322) using tetracyline and kanamycin as the control antibiotics. Bioguided fractionation of the active fraction led to the isolation of the *γ*-butenolide, spiculisporic acid. Structure elucidation was carried out using ^1^H and ^13^C-NMR spectroscopic analysis. The compound showed good antimicrobial activities with minimum inhibitory concentration (MIC) values ranging from 3.9 to 31.25 μg/mL against all tested strains. Gas chromatography coupled to mass spectrometry (GC-MS) profiling was also carried out to identify the metabolites in the microbial crude extract. In conclusion, endophytic fungi, *Aspergillus cejpii*, isolated from *Hedera helix* L. roots showed promising antimicrobial activity which merits further in-depth investigations for potential utilization as a source of new antibiotics in the future. It can also be considered as a novel source for spiculisporic acid.

**Supplementary Information:**

The online version contains supplementary material available at 10.1007/s42770-023-01224-7.

## Introduction

*Hedera helix* L. (*H. helix* L.), Ivy or English Ivy, is a climbing plant belonging to family Araliaceae [1]. The family is commonly known as the ginseng family. Most members are shrubs or trees, with several climbers and a few herbs. It is distributed in Africa, Asia, Europe, Australasia, and Northern America. Phytochemical screening of *H. helix* L. revealed the presence of different chemical constituents including triterpene saponins, phenolic compounds, flavonoids, polyacetylenes, unsaturated sterols, tannins, terpenoids, alkaloids, and carbohydrates [2–7]. The plant is well known for its respiratory, anti-inflammatory, analgesic, immunological, anticancer, antimicrobial, and anti-thrombin activities [7–11].

In the continuous search performed by pharmaceutical industry and researchers for discovering novel products, natural sources were found to be superior for their content of novel substances that have the potential to be developed into new industrial products [12, 13]. Hence, their search for new secondary metabolites should be extended to cover the organisms that inhabit the plant kingdom.

Endophytes are microorganisms that inhabit the interior of plants, especially leaves, stems, and roots residing in the internal tissues of the living plants without causing any immediate apparent negative effects. Almost all classes of plants were found to host one or more endophytes. Throughout the last decade, scientists were able to isolate approximately 6500 endophytic fungi from herbaceous plants and trees in addition to screening them for biological activities [12]. Endophytes are abundant with rich biodiversity and have been discovered in every plant species examined so far. They act as chemical synthesizers inside plants. Many of them can integrate bioactive compounds that can be used by plants for defense against pathogens, and some of these compounds were proved to be useful for novel drug discovery. Up till now, most of the natural products from endophytes were found to be antimicrobial agents, anticancer, and other bioactive compounds by their various functional parts [14].

Infectious diseases caused by bacteria and fungi affect millions of people worldwide. Throughout history, they have continued to be a major cause of death and disability. Today, infectious diseases account for one-third of all deaths in the world; the World Health Organization (WHO) estimates that nearly 50,000 people die each day/year from infectious diseases [15]. Yet, the upsurge of multidrug-resistant pathogenic microorganisms has threatened the clinical efficiency of many current antibiotics. This global issue has provoked a worldwide search for new antibiotics from numerous sources, and one of these most important sources is the endophytic fungi inhabiting the plant kingdom. Recently, researchers have been directing their efforts towards discovering the hidden treasures of endophytic fungi and their assorted metabolites as they are much easier to grow and were found to be a promising source of novel bioactive compounds including antimicrobials [16]. Accordingly, the objective of this work was to isolate, identify, and characterize active compounds produced by the endophytic fungi associated with *H. helix* L. using a bioguided fractionation scheme, which combines analytical procedures with bioassays, representing a prompt and cost-effective method to discover possible useful fractions and pure compounds.

## Materials and methods

### Plant material

*H. helix* L. plant was obtained from Arab Company of Pharmaceuticals and Medicinal Plants (Mepaco-Medifood) El-Sharkya, Egypt, with coordinates near (30.3799° N, 31.4544° E). Plant samples were supplied by Dr. Mokhtar Bishr Technical Director of Mepaco Company.

### Isolation of endophytic fungi from *H. helix* L.

Endophytic fungi were isolated by the method described by [17] with some modifications. Briefly, different parts of *H. helix* L. including leaves, stems, and roots were washed with running water and allowed to air dry. For surface sterilization, the plant parts were dipped twice in 70% ethanol for 2 min., washed multiple times (3 times) with sterile water, then immersed in 0.5% sodium hypochlorite for 1 min., washed again 3 times with sterile water, and allowed to dry. Then, each part was cut into small cubes of 1 mm diameter to release the endophytic fungi and then inoculated aseptically on Potato Dextrose Agar (PDA) (Himedia, India) plates supplemented with 250 μg/mL of both streptomycin and gentamicin to inhibit bacterial growth. Non-inoculated PDA plates were used as negative control in addition to PDA plates inoculated with 1 mL of the last washing water to ensure the inhibition of the surface microorganisms. The plates were incubated for 7 to 21 days at 25 °C. Various mycelia growing out of the pieces were cultured in new PDA plates several times, and the isolated pure fungi were maintained on PDA slants and kept at 4 °C.

### Characterization of isolated endophytic fungi

Morphological characterization of the isolated fungi was performed using a standard taxonomic key that included colony characteristics such as texture, shape, and color [18]. For microscopic examination, promising fungus was cultured on PDA for 7 days using the slide culture method, and hyphae, conidiophore, and conidia were observed under a microscope after staining with lactophenol cotton blue. Preliminary fungus identification was based on both the morphological and microscopical characteristics of the fungus [19].

### Fungi identification through molecular approach

Genomic DNA was extracted by Quick-DNA™ Fungal/Bacterial Microprep Kit (Zymo research #D6007) following the manufacturer’s protocol. ITS 1 and ITS 4 were used as forward and reverse primers, respectively, to amplify internal transcribed spacer ribosomal RNA (ITS rRNA region. PCR was conducted using Maxima Hot Start PCR Master Mix (Thermo; K1051). Thermal cycling conditions were as follows: initial denaturation at 95 °C for 10 min, 35 cycles of denaturation at 95 °C for 30 s, annealing at 57 °C for 1 min, and extension at 72 °C for 1.5 min. The post-cycling expansion was done as one cycle at 72 °C for 10 min. The PCR yields were then purified by GeneJET PCR Purification Kit (Thermo K0701, Waltham, MA, USA) in accordance with the manufacturer’s instructions, and the purified DNA was stored at −20 °C. Finally, the refined PCR products were sequenced using an ABI 3730xl DNA sequencer (Applied Biosystems™, ThermoFisher). DNA extraction, PCR, and PCR products purification were carried out by Sigma Scientific Services Company (Egypt), while DNA sequencing was performed by GATC Company (Washington).

Final sequence of the gene product of the fungal isolates was aligned against available sequences in the GenBank database using NCBI BLAST (Basic Local Alignment Search Tool; http://blast.ncbi.nlm.nih.gov/). The neighbor-joining technique was used to build the phylogenetic tree using the MEGA 5 software. The identified isolates’ sequences were deposited to the GenBank database and assigned accession numbers [20, 21].

### Extraction of the fungi secondary metabolites

Small-scale production was carried out for all isolated fungi for the purpose of preliminary biological screening. Solid rice medium was prepared by adding 100 g of rice mixed with 120 mL of sterilized water in Erlenmeyer flasks (1L) and autoclaved at 121 °C for 20 min. Plugs from PDA pure fungal cultures were used to inoculate three solid rice flasks, and they were allowed to grow for 21 days at room temperature. Fungal metabolites were then extracted using 600 mL of ethyl acetate (EtOAc) for three consecutive times till exhaustion as described by [22], and the extract was evaporated under vacuum yielding a dark brown residue which was reserved for further biological and chemical investigations.

For mass production of the fungal isolate showing the highest antimicrobial activity, fifteen solid rice medium flasks were used following the same procedure.

### Determination of antimicrobial activity of fungal crude extracts

Antibacterial activities of secondary metabolites extracted from *H. helix* L. fungal endophytic isolates were determined by screening the fungal crude extracts against Gram-positive and Gram-negative bacterial reference isolates using MIC microtitre plate assay [23].

Five Gram-negative bacteria viz., *Escherichia coli* (*E. coli*, ATCC 25922), *Pseudomonas aeruginosa* (*Ps. aeruginosa*, ATCC 9027), *Serratia marcescens* (*S. marcescnes*, ATCC 13880), *Acinetobacter baumannii* (*A. baumannii,* ATCC 19606), and *Salmonella typhi* (*S. typhi,* ATCC 6539) and one Gram-positive bacteria *Staphylococcus aureus* (*S. aureus*, ATCC 6538) were used.

All bacterial isolates were cultured overnight in Tryptone Soya Broth (TSB, Himedia, India) sterile medium at 37 °C. The dried crude extracts of the endophyte fungi were dissolved in DMSO in initial concentration of 500 μg/mL. In a sterile 96-well microtiter plate, 100 μL of sterile TSB were added, followed by a two-fold serial dilution of each fungal extract to reach concentrations ranging from 250 to 1.95 μg/mL), then 10 μL of adjusted bacterial inoculum (≈ 10^6^ CFU /mL) were added to each well. Tetracycline was used as a positive control. None inoculated wells were used as negative controls, and wells inoculated with the bacteria but without the extracts were used as positive controls. Microtiter plates were incubated overnight at 37 °C. After incubation, wells were checked for turbidity, and the minimum inhibitory concentration (MIC) values were determined as the lowest concentration that inhibits the growth of bacteria.

### Bioguided fractionation of highly active endophyte’s secondary metabolites

The endophytic fungal extract demonstrating the highest antimicrobial activity was subjected to bioguided fractionation to isolate the bioactive fractions and compounds. Accordingly, the total fungal ethyl acetate fraction of the promising fungus (35 g) was fractionated on a vacuum liquid chromatography (VLC) packed with silica gel 60 mesh (Merck, Darmstadt, Germany). Elution was performed using a gradient system of *n*-hexane:EtOAc mixture (100:0 v/v – 0:100 v/v) followed by DCM:MeOH (100:0 v/v – 0:100 v/v) to yield 6 collective fractions after investigating by TLC under UV light and spraying with vanillin/sulfuric acid reagent. The most active fraction, AC4 (7 g), was then applied again on VLC packed with silica gel 60 mesh, and elution was performed using a gradient system of DCM:MeOH (100:0 v/v − 0:100 v/v). Subfractions were tested, and the most active subfraction, AC4’-1, was further purified on silica columns using an isocratic system of DCM:MeOH (95:5 v/v) to afford a pure compound (**1)** (150 mg).

### Antimicrobial susceptibility of ethyl acetate fractions and purified compound

The antimicrobial activities of the collective fractions, subfractions, and isolated compound were monitored using the six reference strains previously used in this study, in addition to three drug-resistant clinical isolates: *Ps. aeruginosa*, *A. baumannii*, and MRSA. MIC microtitre plate assay was carried out as mentioned previously.

### Structural elucidation of spiculisporic acid

Structural identification of compound 1 was achieved using mass spectroscopy, 1D and 2D NMR. NMR spectra were recorded on a Bruker High Performance Digital FT-NMR-Spectrophotometer Avance III HD (1H-NMR: 400 MHz, 13C-NMR: 100 MHz, Bremen, Germany). NMR spectra were recorded in CD_3_OD using TMS as an internal standard. The solvent used was 500 μl, and the number of scans (NS) was 200 automatically locked based on the CD_3_OD signal at 3.30 ppm. Chemical shift values were recorded in δ ppm.

Mass spectrum was obtained using an XEVO TQD triple quadrupole(Waters Corporation, Milford, MA01757 USA) mass spectrometer. Source temperature 150 °C, cone voltage 30 eV, capillary voltage 3 kV, desolvation temperature 440 °C, cone gas flow 50 L/h, and desolvation gas flow 900 L/h. Mass spectra were detected in the ESI between m/z 100 and 1000. The peaks and spectra were processed using the Maslynx 4.1 software and tentatively identified by comparing their retention times (Rt) and mass spectra with reported data.

### GC-MS analysis

GC-MS analysis was performed on a Shimadzu GCMS-QP 2010 (Tokyo, Japan) with Rtx-5MS capillary column (30 m × 0.25 mm i.d. × 0.25 μm film thickness) (Restek, USA). The capillary column was coupled to a quadrupole mass spectrometer (SSQ 7000; Thermo-Finnigan, Bremen, Germany). The oven temperature was kept at 45 °C for 2 min (isothermal), programmed to 300 °C at 5 °C/min, and kept constant at 300 °C for 5 min (isothermal); the injector temperature was 250 °C. Helium was utilized as a carrier gas with a constant flow rate set at 1.41 mL/min. Diluted samples (1% v/v) were injected with a split ratio of 15:1, and the injected volume was 1 μL. The MS parameters were as follows: interface temperature, 280 °C; ion source temperature, 200 °C; EI mode, 70 eV; and scan range, 35–500 amu. The overall run time was about 30 min. Compounds were identified using retention times and mass spectra matching with the National Institute of Standards and Technology (NIST-11) and Wiley library databases.

### Statistical analysis

All statistics have been conducted using GraphPad Prism 5 (LaJolla, CA, USA) software. The Kolmogorov-Smirnov test was applied to assess the normal distribution of the collected data. Since the data were found to be normally distributed, a two-way analysis of variance (ANOVA) with Bonferroni post-test was utilized to calculate the significant difference between means. *P* < 0.0001 was considered statistically significant.

## Results

### Isolation and identification of cultivable endophytic fungi

In the present study, a total of 6 fungal strains were isolated from the different parts of *H. helix* L. Four isolates originated from roots, one from stems, and one from leaves. The isolated fungi were preliminary identified based on morphological and microscopic characters, and the identification was further confirmed using amplification and sequencing of ITS rRNA gene. The ITS rRNA gene sequences were BLAST aligned and submitted at the GenBank under the accession numbers provided in Table 1.

**Table 1:** Identified endophytic fungal isolates from different parts of *H. helix* L. with accession numbers

Isolated fungus	Plant organ	Accession number
*Aspergillus niger*	Leaf	MW142509
*Saccharomycopsis fibuligera*	Stem	MW165540
*Talaromyces assiutensis*	Root	MW165542
*Aspergillus cejpii*	Root	MW110482
*Talaromyces trachyspermus*	Root	MW131876
*Fusarium solani*	Root	MW209717

### Preliminary antimicrobial screening

The metabolites produced by the endophytic fungal isolates in the ethyl acetate crude extracts were subjected to preliminary antimicrobial activity against various bacterial strains using tetracycline as a positive control. *Aspergillus cejpii* (*A. cejpii*) metabolites showed a promising wide spectrum of antibacterial activity in comparison to other tested endophytic extracts (Table 2). The highest antibacterial activity with the least MIC (62.5 μg/mL) was detected for the ethyl acetate extract of *A. cejpii* against *E. coli*, *Ps. aeruginosa*, *S. marcescnes*, and *S. typhi* followed by *S. aureus* and *A. baumannii* with MIC values of 125 μg/mL. Accordingly, *A. cejpii* was selected for further in-depth investigations.

**Table 2:** MIC values of endophytic fungal isolates’ extracts against different microbial reference strains

Endophytic fungal isolates	Tested bacterial reference strains					
	*E. coli* ATCC 25922	*Ps.* *aeruginosa* ATCC 9027	*S. aureus* ATCC 6538	*S. marcescens* ATCC 13880	*A. baumannii* ATCC 19606	*S. typhi* ATCC 6539
	MIC values (μg/mL)					
*Aspergillus niger*	≥ 250	≥ 250	-	≥ 250	-	≥ 250
*Saccharomycopsis fibuligera*	-	-	-	-	-	≥ 250
*Talaromyces assiutensis*	≥ 250	-	-	≥ 250	-	-
*A. cejpii*	62.5 ± 0.94	62.5 ± 0.30	125 ± 0.36	62.5 ± 0.47	125 ± 0.60	62.5 ± 0.72
*Talaromyces trachyspermus*	≥ 250	≥ 250	-	-	-	-
*Fusarium solani*	-	-	-	-	-	-

*All measurements were done in triplicates, and values are expressed as mean ± SD

*(-) No activity detected

### Characterization of *A. cejpii*

**Fig. 1:**
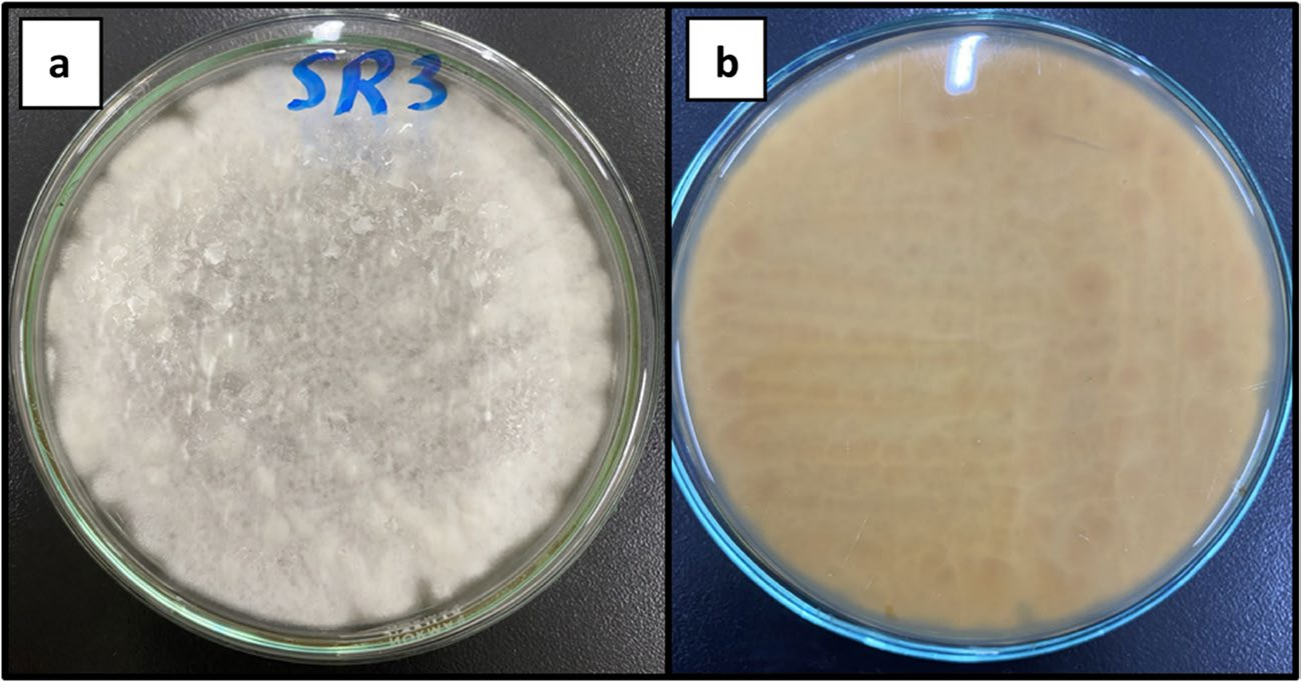
Morphological characters of *A. cejpii* on PDA plate. **a** White downy colony on the surface of the plate, **b** brownish color on the reverse side of the plate

**Fig. 2:**
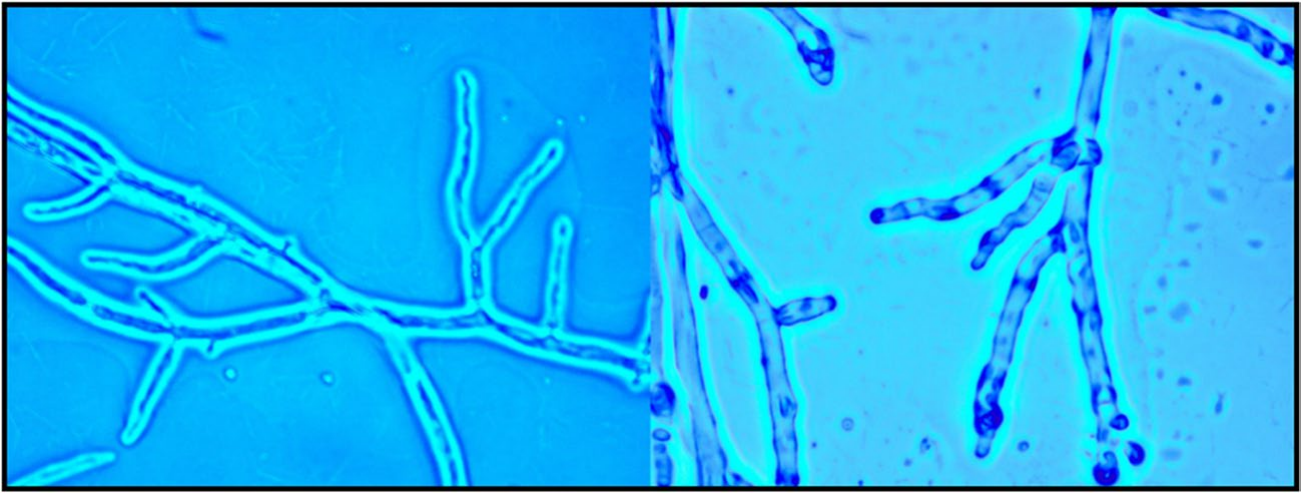
Microscopic examination of *A. ceipii* revealing hyaline, septate hypha with uniserate phialides

**Fig. 3:**
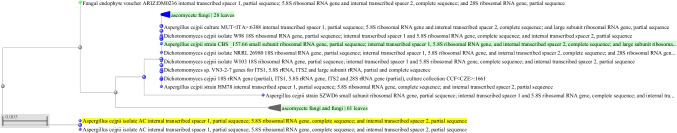
Phylogenetic tree of the isolated endophyte *A. cejpii* produced using BLAST pairwise alignments, NCBI*.* The isolate sequence shows 85% Query coverage, E-value =0, and 96.7% percent identity

Complete identification of *A. cejpii* isolate was performed based on both morphological and microscopical characteristics. Macroscopic description of growing fungi showed fast-growing fungi with white, downy colonies that turned yellowish-white by age, while the reverse side was brownish in color (Fig. 1). Microscopic examination of *A. cejpii* revealed septated, hyaline hyphae with uniseriate phialides, and small round conidia (Fig. 2). Blast sequence analysis of the endophytic fungal isolate revealed 99% similarity with *A. cejpii* (Fig. 3).

### Bioguided fractionation of *A. cejpii* extract

**Fig. 4:**
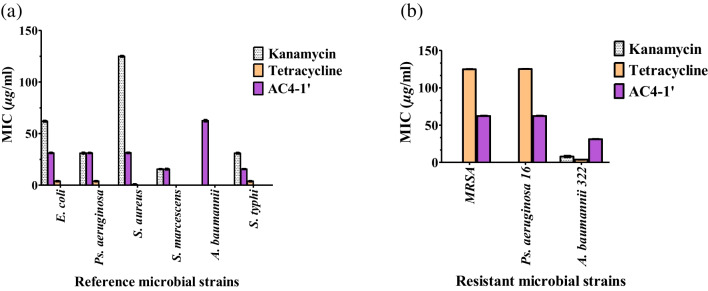
MIC values of the ethyl acetate subfractions of *A. cejpii* against different microbial reference strains (**a**) and different resistant strains (**b**) compared to kanamycin and tetracycline. All measurements were done in triplicates, and values are expressed as means ± SD

**Fig. 5:**
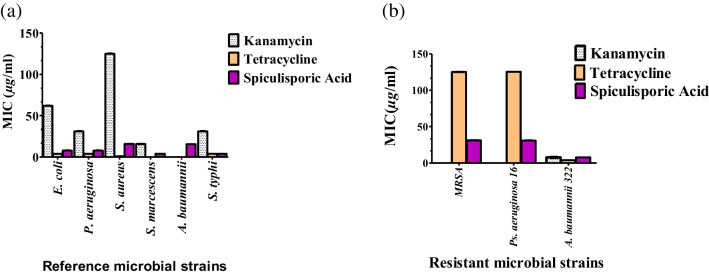
MIC values of spiculisporic acid isolated from the most active subfractions against different microbial reference strains (**a**) and against resistant microbial strains (**b**). All measurements were done in triplicates, and values are expressed as means ± SD

A bioassay-guided fractionation was performed for investigation and identification of the active fractions and metabolites in *A. cejpii* fungus extract (Fig. 1, supplementary material). Fractionation and purification gave 5 subfractions of which fractions AC4-1’ and AC4-2’ showed the highest antimicrobial activity compared to all other subfractions. The activity was tested against previously mentioned Gram-positive and Gram-negative bacterial strains in addition to three drug-resistant clinical isolates viz., MRSA-H1, *Ps. aeruginosa* PS 16, and *A.baumannii* ACT 322 using tetracyline and kanamycin as the control antibiotics. Fractions AC4-1’ and AC4-2’ exhibited similar pattern of activity against *Ps. aeruginosa* and *S. marcescnes* with MIC values of 31.25 μg/mL and 15.63 μg/mL, respectively. Both fractions showed similar MIC values of 62.5 μg/mL against *A. baumannii* as well as resistant strains MRSA and *Ps. aeruginosa.* On the other hand, AC4-1’ showed approximately 50% increase in its antimicrobial activity against *E. coli*, *S. aureus*, and *A. baumannii* when compared to AC4-2’ with MIC value of 31.25 μg/mL. It also showed very promising activity against *S. typhi* where it exhibited MIC value of 15.63 μg/mL (Fig. 4). Upon further fractionation, a major compound was isolated from subfractions AC4-1’, compound **1**. The isolated compound proved to be the most active tested sample with the best MIC values against all tested strains including the drug-resistant ones (Fig. 5), with MIC values ranging from 3.9 to 31.25 μg/mL. Compound **1** showed a very promising activity against resistant bacterial strains: MRSA-H1 and *Ps. aeruginosa* PS 16 where it showed MIC value of 31.25 μg/mL compared to standard drugs kanamycin and tetracycline. It is worthy to mention that kanamycin did not show any activity against both strains, while tetracycline showed weak activity (MIC= 125 μg/mL).

Regarding resistant strain *A. baumannii* 322, compound **1** showed very potent activity with MIC value of 7.8 μg/mL which was comparable to the activity achieved by kanamycin.

MIC values of the subfractions and isolated compound against all tested bacterial strains are presented in Table 3 and illustrated in Figs. 4 and 5.

**Table 3:** MIC values of the subfractions and isolated compound against all tested bacterial strains

Tested fraction	Tested bacterial pathogens								
	*E. coli* ATCC 25922	*Ps. aeruginosa* ATCC 9027	*S. aureus* ATCC 6538	*S. marcescens* ATCC 13880	*A. baumannii* ATCC 19606	*S. typhi* ATCC 6539	MRSA-H1	*Ps. aeruginosa* PS 16	*A. baumannii* ACT 322
	MIC values (μg/mL)								
AC4-1' fraction	31.28 ± 0.055	31.25 ± 0.250	31.35 ± 0.409	15.51 ± 0.461	62.50 ± 1.00	15.61 ± 0.101	62.33 ± 0.764	62.26 ± 0.681	31.25 ± 0.250
Spiculisporic acid	7.80 ± 0.100	7.76 ± 0.252	15.60 ± 0.100	3.90 ± 0.100	15.433 ± 0.208	3.80 ± 0.265	31.133 ± 0.321	30.96 ± 0.451	7.80 ± 0.100
Kanamycin	62 ± 0.500	31 ± 0.557	125 ± 0.500	15.60 ± 0.100	-	31 ± 0.500	-	-	7.800 ± 0.100
Tetracycline	3.893 ± 0.090	3.90 ± 0.100	0.676 ± 0.499	0.050 ± 0.010	-	3.87 ± 0.060	125.06 ± 0.404	125.23 ± 0.493	3.87 ± 0.061

*All measurements were done in triplicates, and values are expressed as mean ± SD

*(-) No activity detected

### Identification and elucidation of spiculisporic acid

Compound **1** was isolated as white needle crystals. Its molecular formula was determined by ESI mass spectrometry as C_17_H_28_O_6_ as it gave strong molecular ion at [M + H]^+^ m/z 329.1962 (calc. for C_17_ H_29_ O_6_^+^: 329.1986). ESI [M-H]^-^ m/z 327.1809 having four degrees of unsaturation. ^1^H- and ^13^C-NMR data assignments of compound **1** were obtained by interpretation of ^1^H–^1^H COSY, HSQC, and HMBC 2D spectra (Figs. S2–6, supplementary material). The ^1^H-NMR spectrum of the compound exhibited a characteristic doublet of doublet signal at ^δ^H 3.03 (dd, H-5). It also displayed signals characteristic for three methylenes [^δ^H 2.68–2.47 (m, H-2 and H-3), 1.92–1.81 and 1.58–1.49 (each m, H-6)], and one methyl [^δ^H 0.91 (t, J= 6.7Hz, CH_3_-15)] in addition to 16 aliphatic protons [^δ^H 1.38 – 1.27 (m, H-7~14)] (Table 4). The ^1^H–^1^H COSY spectrum showed a correlation between H-2 (δ H 2.58) and H-3 (δ H 2.50). The methyl protons at H-15 were coupled with the methylene protons at H-14 (δ H 1.38 – 1.27). Correlations between the methylene protons at H-6 (δ H 1.87, 1.54) and H-5 [δ H 3.03 (dd, J = 10.8, 3.2 Hz)] and H-7 (δ H 1.38–1.27) were also observed.

**Table 4:** ^1^H and ^13^C NMR spectral data of spiculisporic acid from *A. cejpii*

Position	^13^C-NMR spectral data	^1^ H-NMR spectral data
1	176.96	
2	27.45	2.58
3	29.08	2.50
4	86.59	
4- COOH	172.53	
5	50.94	3.03 (dd. J = 10.8, 3.2 Hz)
5- COOH	173.93	
6	27.66	1.86 (m), 1.54 (m)
7	27.45	1.27–1.38 (m) ^(b)^
8	29.08	1.27–1.38 (m) ^(b)^
9	29.00	1.27–1.38 (m) ^(b)^
10	29.17	1.27–1.38 (m) ^(b)^
11	29.28	1.27–1.38 (m) ^(b)^
12	29.33	1.27–1.38 (m) ^(b)^
13	31.68	1.27–1.38 (m) ^(b)^
14	22.36	1.27–1.38 (m) ^(b)^
15	13.13	0.91 (t)

^a,b^Overlapping signals

Measured in CD_3_OD

**Fig. 6:**
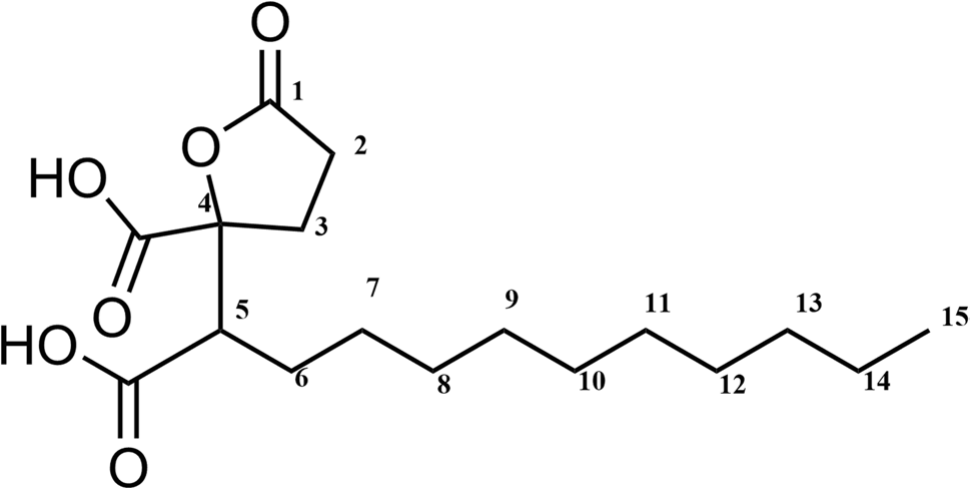
Structure of spiculisporic acid isolated from *A. cejpi*

^13^C- NMR spectra revealed 3 carbonyl signals at ^δ^C 176.96, 173.93, and 172.53 which suggested that this compound is a tricarboxylic acid derivative, in addition to signals corresponding to one quaternary carbon (^δ^C 86.59), one methine (^δ^C 50.94), eleven methylenes (^δ^C 31.68–22.36), and one methyl (^δ^C 13.13) (Table 4). In the HMBC spectrum, the signal at ^δ^H 3.03 revealed a cross-peak with ^δ^C 173.93, also signals at ^δ^H 2.50 and 2.58 showed cross-peaks with ^δ^C 50.94, 172.53, and 176.95 confirming the position of the proposed carbonyls within the chemical structure. From the above data, compound (**1**) was identified as spiculisporic acid with molecular formula C_17_H_28_O_6_ (Fig. 6). The NMR data of the compound was similar with that previously isolated from *T. trachyspermus* [24].

### GC-MS analysis

GC-MS analysis of the ethyl acetate extract of *A. cejpii* resulted in the identification of a total of 36 compounds with a total area percentage of 93.94%. The identified compounds belonged to various classes of compounds including aliphatic hydrocarbons, fatty acids, fatty acids esters, and fatty alcohols. The mass spectra of the compounds were compared with data from NIST and Wiley libraries, and the peaks were characterized and identified.

Hydrocarbons were the predominant class of identified compounds representing 40.5% followed by fatty acids and their derivatives including ethyl esters, alcohols, ketones, and ethers, representing 21.94%. The prevailing hydrocarbons were *n*-pentatriacontane (8.93%), *n*-tetratriacontane (6.39%), 17-pentatriacontene (3.06%), and tetracosane (2.83%). Regarding fatty acids and their derivatives, *n*-hexadecanoic acid (4.67%) was the major identified fatty acid. (9Z,12Z)-9,12-Octadecadien-1-ol (4.63 %) and its acetate, linoleyl acetate (4.76%), were also found in abundance in the extract. The isolated compound spiculisporic acid was detected in the total ethyl acetate extract with an area percentage of 0.69%. The retention time (Rt) of the identified compounds along with their molecular formula (MF), molecular weight (Mwt), and concentration (Area %) are presented in Table 5.

**Table 5:** Compounds identified in ethyl acetate extract of *A. cejpii via* GC-MS

Peak #	Compound	MW	Molecular formula	Rt. time (min)	Area %
1	5-Methyltetradecane	212	C_15_H_32_	33.419	0.61
2	(1S,2E,6E,10R)-3,7,11,11-Tetramethylbicyclo [8.1.0] undeca-2,6-diene	204	C_15_H_24_	34.099	2.46
3	*n*-Hexadecanoic acid	256	C_16_H_32_O_2_	34.351	4.67
4	Hexadecanoic acid, ethyl ester	284	C_18_H_36_O_2_	34.982	0.62
5	1,3,6,10-Cyclotetradecatetraene, 3,7,11-trimethyl-14-(1-methylethyl)-, [S-(E,*Z*,E,E)]-	272	C_20_H_32_	35.303	1.13
6	*n*-Eicosane	282	C_20_H_42_	35.438	1.15
7	2,4-Dimethylicosane	310	C_22_H_46_	37.364	2.38
8	(9Z,12Z)-9,12-Octadecadien-1-ol	266	C_18_H_34_O	37.591	4.63
9	2-Hydroxycyclopentadecanone	240	C_15_H_28_O_2_	37.737	2.31
10	3 E,E,Z-1,3,12-Nonadecatriene-5,14-diol	294	C_19_H_34_O_2_	38.080	1.72
11	Hexadecanenitrile	237	C_16_H_31_N	38.246	0.95
12	1-Heptacosanol	396	C_27_H_56_O	40.335	2.90
13	Hexadecyl cyclohexane	308	C_22_H_44_	40.525	1.33
14	2-Octyl-1-dodecanol	298	C_20_H_42_O	40.978	0.50
15	Docosyl ethyl ether	354	C_24_H_50_O	41.169	0.76
16	Carbonic acid, decyl hexadecyl ester	426	C_27_H_54_O_3_	42.213	0.83
17	*n*-Heptadecylcyclohexane	322	C_23_H_46_	42.315	3.35
18	Nonyl tetradecyl ether	340	C_23_H_48_O	42.655	0.71
19	*n-*Tetracosane	338	C_24_H_50_	42.843	2.83
20	1,4-dimethyl-2-octadecyl-Cyclohexane	364	C_26_H_52_	43.155	0.75
21	Sulfurous acid, cyclohexylmethyl tridecyl ester	360	C_20_H_40_O_3_S	43.314	3.13
22	2-Ethylhexadecane	254	C_18_H_38_	43.445	1.43
23	*n*-Tetratriacontane	478	C_34_H_70_	43.733	6.39
24	Unknown	-	-	44.463	3.62
25	Sulfurous acid, cyclohexylmethyl pentadecyl ester	388	C_22_H_44_O_3_S	44.975	1.71
26	9,12-Octadecadien-1-ol, acetate (Linoleyl acetate)	308	C_20_H_36_O_2_	45.063	4.76
27	*n*-Pentatriacontane	492	C_35_H_72_	45.341	8.93
26	Heneicosylcyclohexane	378	C_27_H_54_	45.653	0.99
27	17-Pentatriacontene	490	C_35_H_70_	45.978	3.06
28	Spiculisporic acid	328	C_17_H_28_O_6_	46.182	0.69
29	Hexatriacontane	506	C_36_H_74_	46.398	1.93
30	Diglycolic acid, decyl phenethyl ester	378	C_22_H_34_O_5_	46.550	5.96
31	Hexatriacontane	506	C_36_H_74_	46.845	1.93
32	1,6,10,14,18,22-Tetracosahexaen-3-ol	426	C_30_H_50_O	48.984	1.45
33	Hydratropic acid, tridec-2-yn-1-yl ester	328	C_22_H_32_O_2_	49.084	5.62
34	Glutaric acid, 2-methylpent-3-yl phenethyl ester	320	C_19_H_28_O_4_	49.183	3.49
35	Benzoic acid, 4-amino-, 4-hydroximino-2,2,6,6-tetramethyl-1-piperidinyl ester	305	C_16_H_23_N_3_O_3_	53.023	3.08
36	Unknown	-	-	55.673	1.16
37	9 β, 10α-Pregn-4-ene-3,20-dione	314	C_21_H_30_O_2_	56.194	2.45

## Discussion

During recent years, a constant increase in global health problems caused by drug-resistant bacteria and fungi has been observed. Different processes are included in antibiotic resistance such as changes in antibiotic permeability, changes in target molecules, and enzymatic breakdown of medicines. Recently, the number of new antibiotics created has dropped considerably leading to the inevitability of a broader search for new and effective antimicrobial agents. Nowadays, traditional medicinal plants are expected to provide more promising antimicrobial agents.

Endophytes have been recognized as useful sources of bioactive secondary metabolites in addition to producing many bioactive compounds beneficial to pharmaceuticals, environment, agriculture, and industries [25]. According to several studies, numerous endophytic fungi have the ability to produce antimicrobial substances with broad-spectrum bioactivity [26–31]. Thus, our goal was to search for compounds of a natural microbial source with promising antimicrobial activities. In this study, we examined all parts of *H. helix* L*.* plant for isolation of endophytic fungi with antimicrobial activities, where only those belonging to genus *Aspergillus* isolated from the root showed promising antimicrobial activity against a panel of Gram-positive and Gram-negative bacteria.

Genus *Aspergillus* represents a broadly spread genus of fungi that are widely known for possessing potent medicinal potential including antimicrobial, cytotoxic, and antioxidant activities which is highly credited to its rich content of secondary metabolites [32].

Accordingly, a study was designed based on bioguided fractionation to specify the active compound in *A. cejpii* with the best antimicrobial activity. In our study, solid state fermentation medium using complex solid rice was used as standard condition for both small-scale and large-scale production to produce sufficient quantities of the compounds of interest. Selection of solid-state media was based on an earlier study reporting that the cultures grown on solid media generated extracts with masses of one to two orders of magnitude larger than the same fungus grown in any of the liquid media [33].

The ethyl acetate fungal extract of *A. cejpii* showed significant broad-spectrum activity against Gram-positive and Gram-negative bacteria. To the best of our knowledge, this is considered the first report for the isolation of *A. cejpii* from the roots of *H. helix* L. However, several studies have been reported on the isolation and antibacterial activity of different species of endophytic fungi belonging to genus *Aspergillus*, and the results were in accordance with our obtained data regarding antimicrobial activity [34, 35].

Further investigations were carried out on the ethyl acetate fractions and subfractions to determine the antibacterial activity of *A. cejpii* secondary metabolites against resistant bacterial strains including MRSA, *Ps. aeruginosa* (PS 16), and *A. baumannii* (ACT 322). *S. aureus* is one of the major causes of community- and healthcare-associated infections. It causes various infections ranging from superficial skin and soft tissue infections to invasive infections, sepsis, and death. MRSA has long been acknowledged as a pathogen associated with healthcare settings. It is considered one of the most dangerous causes of hospital-acquired infections that are becoming increasingly difficult to combat because of emerging resistance to all current antibiotics [36]. *Ps. aeruginosa* is naturally resistant to a variety of antimicrobials and can develop resistance during anti-pseudomonal chemotherapy both of which compromise treatment of infections caused by this organism. It is also a well-known nosocomial pathogen that is considered a serious threat due to its high mortality rate associated with infections. This is attributed to the organism’s high resistance to many antimicrobials and the increase of multidrug resistance in healthcare settings [37–39]. *A. baumannii* is an opportunistic pathogen that is responsible for over 10% of infections occurring in hospitals’ intensive care units. It has the ability to cause serious respiratory tract infections, necrotizing fasciitis as well as infections associated with intravascular devices, ulcers, surgical sites, and severe wounds. Patients infected with *A*. *baumannii* have a mortality rate ranging from 20 to 50%, which is attributed to multiple reasons including cellular functions such as capsule production, biofilm formation, the ability to cope with environmental stress, and the acquisition and expression of multidrug resistance (MDR) genes [40].

Our results demonstrated that the crude extract of *A. cejpii* has a broad-antimicrobial spectrum activity suppressing various bacteria species with MICs ranging from 62.5 to 125 μg/mL against different tested strains compared to other endophytic fungi isolated from the different parts of *H. helix* L. Furthermore, bioguided fractionation of the *A. cejpii* crude extract resulted in isolation, purification, and identification of spiculisporic acid. The compound or its derivatives were previously isolated from several fungi viz., *Aspergillus* sp. [41] *Aspergillus candidus* [42], and *Talaromyces trachyspermus* [24]. Our study is considered the first research to obtain spiculisporic acid from endophytic fungi *A. cejpii* with very promising antimicrobial activities.

Spiculisporic acid isolated from *A. cejpii* exhibited significant promising broad-spectrum antimicrobial activities, with MICs much lower than those obtained by the total extract. The isolated compound achieved significant high activity against drug-resistant Gram-positive bacteria, MRSA, and *Ps. aeruginosa* (PS 16) with MIC value of 31.25 μg/mL which is considered a very promising result compared to tested positive standards, kanamycin which showed no activity against the tested resistant strains and tetracycline with MIC value of 125 μg/mL. Our results came in agreement with a previous study that reported a high antibacterial activity for endophytic *A. cejpii* from *Nelumbo nucifera* against MRSA [43]. In addition, spiculisporic acid exhibited significant high activity against *A. baumannii* (ACT 322), where it showed similar antimicrobial activity to that of kanamycin with MIC value of 7.8 μg/mL. Similar results were observed in a previous study where spiculisporic acid derivatives viz., spiculisporic acids B–D isolated from the culture of *Aspergillus* sp. showed promising antibacterial activities against *S. aureus* ATCC 51650 with inhibition zones of 9.6, 11.6, and 11.5 mm at 20 mg/mL [41].

On the other hand, another group of researchers isolated spiculisporic acid E compound from the culture of the marine-sponge associated fungus *Talaromyces trachyspermus* and evaluated its antimicrobial activity against Gram-positive (*S. aureus* ATCC 25923 and *Bacillus subtilis* ATCC 6633) and Gram-negative (*E. coli* ATCC 25922 and *Ps. aeruginosa* ATCC 27853) bacteria, *Candida albicans*, and MDR isolates from the environment; however, it showed no activity at the highest concentration tested (256 μg/mL). In another study, two antibiotic spiculisporic acid analogues, identified as spiculisporic acid F and G, which were isolated through bioactivity-guided fractionation from the fermentation broth of the sea urchin-derived *Aspergillus candidus*, also displayed antibacterial activity against Gram-negative *Ps. solanacearum* and Gram-positive *S. aureus* [42].

GC-MS analysis of the crude ethyl acetate extract of *A. cejpii* revealed the presence of numerous compounds of various classes with hydrocarbons being the predominant class followed by fatty acids and their derivatives. Identified compounds were reported to possess numerous biological activities including antimicrobial activities. *n*- Pentatriacontane, the major identified hydrocarbon (8.93%), was reported to display antimicrobial potential against fungal pathogens *Mycogone perniciosa* and *Rhizoctonia solani*, as well as against thc bacteria *Bacillus megaterium*, *E. coli*, and *Ps. fluoresens* [44]. *n*-Tetratriacontane, the second most abundant hydrocarbon (6.39%), was previously identified from the methanolic extract of a medicinal plant *Plantago lanceolata* showing excellent antibacterial activities against *E. coli* and *Bacillus cereus* [45]. Tetracosane was also reported to possess antibacterial activity against *S. aureus* and *S. epidermidis* as well as antifungal activity against *Aspergillus niger* and *Bacillus cinerea* [46]. *n*-Hexadecanoic acid (4.67%) and its ethyl ester in addition to spiculisporic acid (0.69%) were found as major components in the extract. Fatty acids play an important role in the formation of lipids in plants, animals, and microorganisms. There have long been reports on the antibacterial activity of fatty acids against both Gram-positive and Gram-negative pathogens. According to previous studies, *n*-hexadecanoic acid has been shown to possess strong antifungal activity towards several human and phytopathogens through inhibition of spore germination or mycelial growth by disruption of plasma membrane [30]. In a research analyzing the essential oil of the leaves *Solanum spirale* Roxb., *n*-hexadecanoic acid was the major component in the essential oils, and it was found to have significant antibacterial activity against both Gram-negative *E. coli* and Gram-positive *S. aureus* [47]. Additionally, derivatives of spiculisporic acid including spiculisporic acids B–D, F, and G isolated from sea urchin-derived fungus *Aspergillus sp.* and *Aspergillus candidus* strain exhibited significant inhibitory activity against *P. solanacearum* and *S. aureus* which was higher than that achieved by the tested positive controls (streptomycin sulfate and kanamycin) [42]. Thus, GC-MS analysis confirms the presence of a number of antimicrobial chemical compounds in the ethyl acetate crude fungal extract, the synergetic effect of which can be credited for the observed promising antimicrobial activity.

## Conclusion

Endophytes are considered a hidden gem that is required to be explored to find new and promising secondary metabolites with diverse biological activities. Our study focused on isolating different fungi from the various organs of the well-known medicinal plant *H. helix* L. The endophytic fungus *A. cejpii* was isolated from the roots of *H.helix* . The fungal extract and spiculosporic acid isolated compound exhibited a broad-spectrum antimicrobial activity against several Gram-positive and Gram-negative strains as well as showing promising activity against the resistant ones. Spiculosporic acid may be used alone or in combination with currently available antibiotics to augment their effects, leading to the improvement of the efficacy against resistant microorganisms. Additional studies are recommended to reveal other biological activities of endophytic fungi hidden in the various organs of the plant as well as further profiling of their secondary metabolites and their mechanism of action.

The outcomes of this study are considered very promising for the discovery of new secondary metabolites with broad-spectrum antimicrobial activity as these findings indicated that the fungus isolated from natural plants could be used as natural agents for pathogenic bacteria bio-control which could offer a natural solution in the pharmaceutical field to fight this bacterial infection.

### Supplementary information

ESM 1 (DOCX 374 kb)
