# Supplementary material for: Antimicrobial activity of spiculisporic acid isolated from endophytic fungus Aspergillus cejpii of Hedera helix against MRSA
Source: Braz J Microbiol. 2024 Jan 17;55(1):515–27. doi: 10.1007/s42770-023-01224-7 (PMC10920557; doi:10.1007/s42770-023-01224-7)
Supplement: Supplementary file 1 — (DOCX 374 kb) [file 42770_2023_1224_MOESM1_ESM.docx]

**Supplementary Material**

**Journal Name: Brazilian Journal of Microbiology**

**Antimicrobial Activity of Spiculisporic Acid isolated from Endophytic Fungus *Aspergillus cejpii* of *Hedera helix* against MRSA**

**Sarah Osama^1^, Moshera El Sherei^2^, Dalia A. Al-Mahdy^2, 3^*, Mokhtar Bishr^4^, Osama Salama^1^ ^†^** **and Marwa M. Raafat^5^***

**Sarah Mohamed Osama^1^,** *Pharmacognosy and Medicinal Plants Department, Faculty of Pharmacy, Future University in Egypt,* (Co-Corresponding author), sara.mohamed@fue.edu.eg

**Moshera El Sherei^2^,** *Department of Pharmacognosy, Faculty of Pharmacy, Cairo University, Egypt,* moshera.elsherei@pharma.cu.edu.eg

**Dalia Adel Al-Mahdy^2, 3^*^,^** *Department of Pharmacognosy, Faculty of Pharmacy, Cairo University, Egypt,* (Corresponding author), dalia.almahdy@pharma.cu.edu.eg, Scopus ID 36141890100.

**^3^***Department of Pharmacognosy and Medicinal Plants, Faculty of Pharmacy, Modern University for Technology and Information*

**Mokhtar Bishr^4,^** *Arab Company for Pharmaceuticals and Medicinal Plants (Mepaco), Egypt***,** mbishr_2000@yahoo.com

**Osama Salama^1†^,** *Pharmacognosy and Medicinal Plants Department, Faculty of Pharmacy, Future University in Egypt,*

**Marwa M. Raafat^5*,^** *Microbiology and Immunology Department, Faculty of Pharmacy, Future University in Egypt, Cairo 11835, Egypt,* marwa.mahmoud@fue.edu.eg. ORCID 0000-0001-5614-5127

*^1^Pharmacognosy and Medicinal Plants Department, Faculty of Pharmacy, Future University in Egypt,* *^2^Department of Pharmacognosy, Faculty of Pharmacy, Cairo University, Egypt,* ^3^*Department of Pharmacognosy and Medicinal Plants, Faculty of Pharmacy, Modern University for Technology and Information, ^4^Arab Company for Pharmaceuticals and Medicinal Plants (Mepaco), Egypt. ^5*^ Microbiology and Immunology Department, Faculty of Pharmacy, Future University in Egypt, Cairo 11835, Egypt*

**Content**

**Fig. (S1): Schematic diagram showing the fractionation of total ethyl acetate fungal extract**

**Fig. (S2): ^1^HNMR spectrum of Spiculisporic acid**

**Fig. (S3): ^13^CNMR spectrum of Spiculisporic acid**

**Fig. (S4): ^1^H- ^1^H COSY spectrum of Spiculisporic acid**

**Fig. (S5): HMBC spectrum of Spiculisporic acid**

**Fig. (S6): HSQC spectrum of Spiculisporic acid**


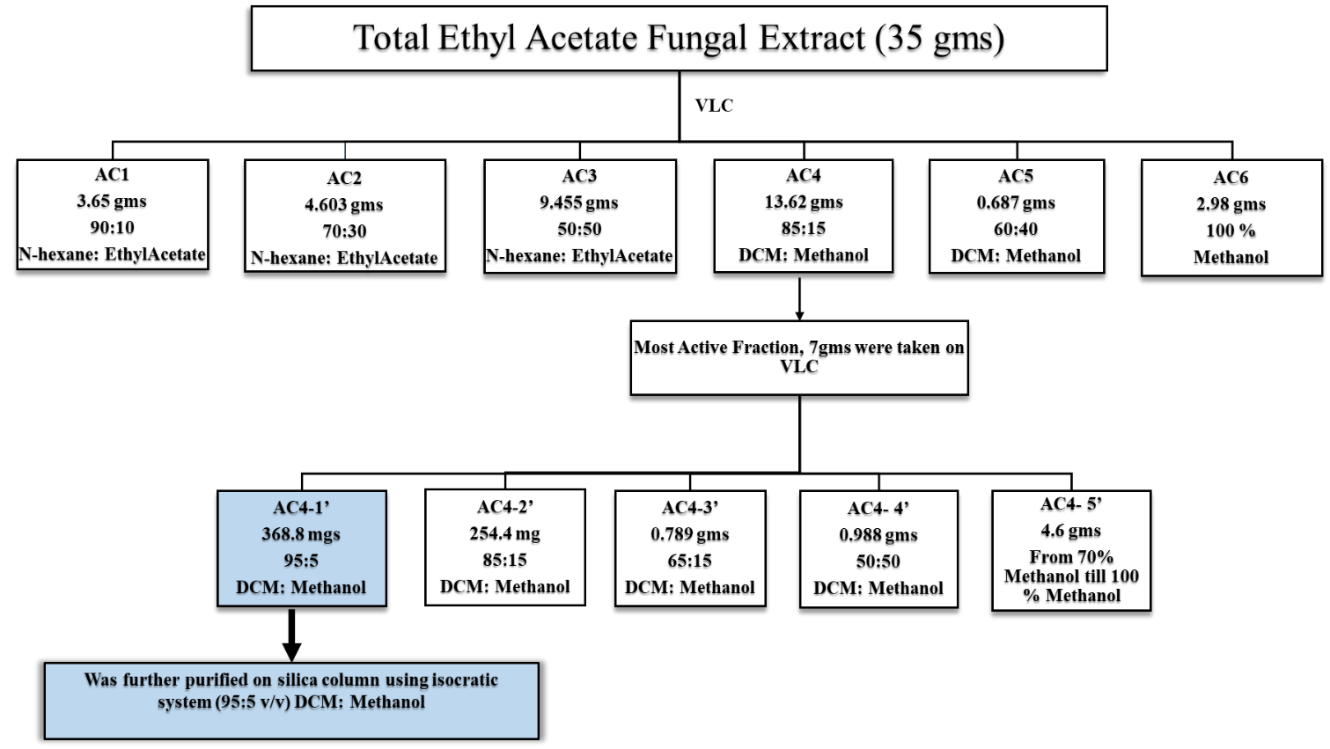


**Fig. (S1): Schematic diagram showing the fractionation of total ethyl acetate fungal extract**

**Fig. (S2): ^1^HNMR spectrum of Spiculisporic acid**


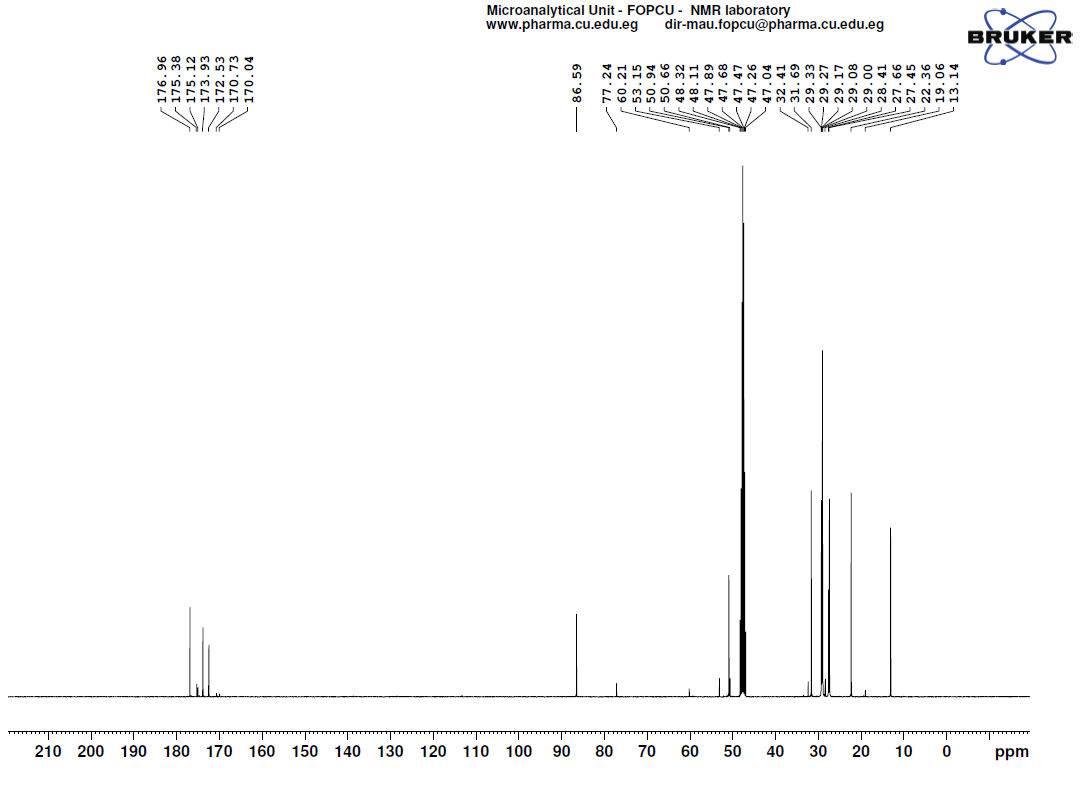


**Fig. (S3): ^13^CNMR spectrum of Spiculisporic acid**

**Fig. (S4): ^1^H- ^1^H COSYspectrum of Spiculisporic acid**

**Fig. (S5): HMBC spectrum of Spiculisporic acid**

**Fig. (S6): HSQC spectrum of Spiculisporic acid**
